# Impact of discontinuing non-pharmacological interventions on cognitive impairment in dementia patients by COVID-19 lockdown. A pilot observational, longitudinal, retrospective study carried out in an adult day center in Spain during the COVID-19 pandemic

**DOI:** 10.3389/fmed.2023.1204151

**Published:** 2023-07-28

**Authors:** Leticia Sánchez-Valdeón, Laura Bello-Corral, Agustín Mayo-Iscar, Diego Fernández-Lázaro, Jesús Seco-Calvo

**Affiliations:** ^1^Department of Nursing and Physical Therapy, University of León, León, Spain; ^2^Department of Statistics and Operations Research and IMUVA, University of Valladolid, Valladolid, Spain; ^3^Department of Cellular Biology, Genetics, Histology and Pharmacology, Faculty of Health Sciences, University of Valladolid, Campus of Soria, Soria, Spain; ^4^Neurobiology Research Group, Faculty of Medicine, University of Valladolid, Valladolid, Spain; ^5^Institute of Biomedicine (IBIOMED), Universidad de León, León, Spain; ^6^Department of Physiology, University of the Basque Country, Leioa, Spain

**Keywords:** dementia, COVID-19, Mini-Mental State Examination (MMSE), day center, non-pharmacological interventions, lockdown

## Abstract

**Background:**

The lockdown imposed during the COVID-19 pandemic led to social isolation and prevented patients with dementia from receiving a suite of non-pharmacological interventions (NPIs) that prevent cognitive decline. This discontinuation of NPIs could substantially affect the mental health status of people with dementia in social care settings, such as adult day care centers (ADCs).

**Propose:**

The study aimed to evaluate the effects of the COVID-19 lockdown on mental health and cognitive impairment in patients with dementia who could not attend their usual ADCs and did not receive our NPIs, based on World Health Organization (WHO) Guidelines.

**Methods:**

Observational, longitudinal, retrospective study carried out in an adult day center in Spain and reported it in accordance with the Strengthening Reporting of Observational Studies in Epidemiology (STROBE) statement. Cognitive status was assessed using the Mini-Mental State Examination (MMSE) in 80 patients attending the ADC of the “Leonese Association of Dementia Patients” (León, Spain), who had been evaluated with this instrument before the COVID-19 lockdown.

**Results:**

We observed a 0.4-point decrease in MMSE score/month (IQR = 1.4) during lockdown versus a 0.1-point decrease/month (IQR = 0.3) before this period (*p* = 0.038). Notably, this translated to >10-point decreases in MMSE score/year in 33.8% of participants during lockdown versus 5.5% earlier (*p* < 0.001). No statistically significant associations (*p* < 0.05) were found between the individual characteristics of the caregivers and the occurrence of the event.

**Conclusion:**

The reported declines in MMSE scores reveal a significant acceleration of cognitive decline during the period of inactivity. This could suggest that our NPIs, focused on slowing cognitive decline, are beneficial and, therefore, necessary in patients with dementia.

## 1. Introduction

Dementia is generally a chronic clinical syndrome that includes a group of specific degenerative diseases characterized by a marked decline in higher brain functions, such as memory, cognitive capacity, and behavior (1). Dementia causes, in patients, dependence on basic activities of daily living and social and occupational functioning, causing patients to become dependent (2). Globally, around 9.9 million people develop dementia every year, with approximately 63% of cases being observed in low- and middle-income countries (3), and in 2021, there were a total of 55 million people diagnosed with dementia worldwide (4). The annual incidence of dementia ranges from 5 to 10 cases per 1,000 among people 64–68 years of age and 40–60 cases per 1,000 among those aged 80–84 (5). Its prevalence is under 2% in 65–69-year-olds but doubles every 5 years, reaching 10–17% among 80–84-year-olds (5). The World Health Organization (WHO) predicts that in 2030 there will be 78 million people with dementia, this rising to 139 million by 2050 (3). In our setting, the Spanish Statistics Institute estimates that the number of people in Spain with dementia will rise to around 600,000 by 2030 and 1 million by 2050 (5). Given these worrying projections for prevalence and incidence, dementia can be considered one of the greatest challenges for primary health and social care worldwide (1, 6).

Various neurodegenerative disorders and factors contribute to the development of dementia through a progressive and irreversible loss of neurons and brain functioning. Currently, there is no cure for any type of dementia (1). The most common type of dementia is Alzheimer’s disease, accounting for two-thirds of all cases (7), followed by vascular and Lewy body dementia (1). Other forms include mixed dementia, which is commonly seen in elderly adults and in which patients have features of more than one type of dementia, and frontotemporal dementia, which is generally uncommon but more common than other types before old age (>65 years) (8). Dementia affects millions of people and is more common as people grow older (about one-third of all people age 85 or older may have some form of dementia) but it is not a normal part of aging. Many people live into their 90s and beyond without any signs of dementia (1, 4). Dementia develops over the years, from an onset that is considered “presymptomatic phase,” also called preclinical phases of the disease, but in which there are pathological changes which might cause short-term memory loss, and personality or behavioral changes, to overt clinical disease, characterized by not being able to plan or carry out complex tasks (9).

There is no single test for diagnosing dementia (10). The initial evaluation requires the assessment of personal health records as well as cognitive and neurological testing (11). For achieving a diagnosis, health records are an important tool, in that they may help identify a decline in cognitive capacity over time (12). A clinical history should be taken by interviewing both patients themselves, some reporting forgetfulness and others failing to remember details of their medical record or having anosognosia, and close family members (11). However, Cognitive testing provides objective evidence of the presence, severity, and nature of cognitive decline (11, 13). Cognitive Tests should be carried out individually and without family members present, to avoid the patient being distracted (13). There are different types of cognitive tests, with short questionnaires being useful for detecting dementia, while tests covering various domains are more effective for confirming the clinical diagnosis of dementia (10). In this way, the Mini-Mental State Examination (MMSE), developed by Folstein in 1975, is one of the most widely used screening tools for cognitive impairment, and scores are easily influenced by education level. MMSE is poor at identifying early dementia, especially mild cognitive impairment. The MMSE consists of six aspects and a total of 30 questions. MMSE is one of the most widely used cognitive function assessment scales in a clinic and has been translated for use in numerous countries (14–16).

The pandemic of coronavirus disease 2019 (COVID-19) caused by the SARS-CoV-2 virus has a considerable impact on people with dementia. Dementia patients are more likely to have adverse medical outcomes with high rates of mortality. In the brain, SARS-CoV-2 has neuroinvasive and neurotropic features with acute and chronic neurovirulent potential (17). Also, dementia patients have also been affected by the measures like isolation and social distancing put in place to control the outbreak, which continue to alter the daily routines of patients and their caregivers (18). Therefore, home confinement is a hostile experience, due to the restrictions on physical, cognitive, and social activities. In our setting, the routines of elderly adults have been disrupted by the cancelation of group activities and the care provided in day centers, which were closed for 3 months, forcing these individuals to stay at home without any care support (19). During confinement due to COVID-19, changes in routines not only affect the person with cognitive impairment, but also determine an overload for the caregiver. The absence of activities of the therapeutic teams, which suspended care services during the quarantine, affects the task of the caregiver and limits her free time (20). This double stress—that of caring for a person with dementia in a context of fear of infection—generates more anxiety and less tolerance for the affected person in some caregivers (21). Other effects of confinement by COVID-19 were anxiety, depression and loneliness suffered by caregivers of patients with dementia (22).

Adult day service centers, such as care homes or adult day care centers (ADCs), provide people with dementia with integrated health and social care for some hours of the day. Several studies have reported the quality of life and care of patients with dementia being better among those who do than those who do not attend ADCs (23, 24). Further, such centers reduce informal caregiver burden and have a positive effect on patients who attend frequently, mainly due to the clinical effectiveness of cognitive therapies they receive (25). These cognitive therapies would encompass a set of non-pharmacological interventions (NPI) that are any non-chemical intervention that is implemented for the benefit of the patient. NPIs, predating modern pharmacology, have been used to improve quality of life, delay deterioration, or relieve pain (26). Cognitive therapies provided in ADCs by health and social workers working with dementia patients and service users in a face-to-face setting have benefits (23, 24) such as: increasing their subjective well-being, mood, and perceived personal satisfaction; reducing anxiety; preventing or improving symptoms of depression; enhancing their sense of self-esteem and social inclusion; and most importantly, improving mental capacity by preserving and most crucially modulating or strengthening cognitive function (24, 25). NPIs are recommended by WHO as tools for reducing the risk of cognitive impairment and dementia with multisectoral interventions (4, 8). Liang et al. (27) have described that NPIs had statistically significant benefits for cognitive function, with Integrated Therapy being the best option for patients with dementia. These authors (28) have suggested that cognitive stimulation and cognitive training, as NPIs, in older adults with mild cognitive impairment significantly improved cognitive function (28). In addition, cognitive training could be the most appropriate method to improve cognitive function in Alzheimer’s patients (29). Moreover, NPIs could reduce psychological symptoms of dementia and maintain or slow decline in cognitive function on psychotic, affective and behavioral subdomains in older adults (26, 30).

However, we hypothesized that the temporary absence, as in confinement during COVID-19, of these NPI could substantially deteriorate the mental health status of dementia patients in social care settings, for example, ADCs. Due to all the above, we assessed, using the MMSE, the effects produced by the COVID-19 pandemic on mental health and cognitive impairment in patients with dementia who were unable to attend their usual ADCs and did not receive NPI as they had been doing until the enforcement of pandemic-related restrictions.

## 2. Materials and methods

### 2.1. Experimental design

A single-center epidemiological, observational, longitudinal retrospective study was carried out in “Leonese Association of Dementia Patients” (Leon, Spain) and report it here in accordance with the Strengthening the Reporting of Observational Studies in Epidemiology (STROBE) statement (31). The sample consisted of all users (*n* = 113) attending the ADC of the “Leonese Association of Dementia Patients” (Leon, Spain). The study was approved by the Ethics Committee of the University of León (Spain) with PI No. 2020–13 “For a tomorrow without Alzheimer’s,” of Leonese Association of Dementia Patients (Spain). All subjects provided written informed consent, in accordance with the Declaration of Helsinki and the 2013 Fortaleza revision (32).

### 2.2. Inclusion criteria

The cohort consisted of ≥18-year-old dementia patients attending the ADC “Leonese Association of Dementia Patients.” All study participants met the following criteria: (i) Diagnosed with Alzheimer’s disease, vascular dementia, Lewy body dementia, and/or frontotemporal dementia*; (ii) Attending the ADC “Leonese Association of Dementia Patients” for at least 1 year; (iii) Receive cognitive stimulation therapy for a continuous period ≥8 months in ADC “Leonese Association of Dementia Patients”; (iv) Completion of the MMSE questionnaire at two points in the study: (a) in the months prior to COVID-19 confinement, in the months of January and February 2020; (b) in the months after COVID-19 confinement, in the months of July and August 2020.

* *All participants were diagnosed by neurology specialists at the León Assistance Complex (León Hospital) based on the clinical criteria and diagnostic protocols established by the Autonomous Community of Castilla-León (Spain).*

### 2.3. Mini-Mental State Examination

We have used the version of the MMSE questionnaire validated by Burke et al. (33) for the Detection of Dementia Spectrum Disorders in Spanish-Speaking Populations. The MMSE, is the questionnaire most used for diagnosing dementia, and to a lesser extent, cognitive decline, and delirium. So, MMSE scores were used to explore potential changes in cognitive functioning. This questionnaire has a sensitivity of 89% and a specificity of 81% for identifying dementia. It takes around 7 min to complete for a person with dementia: The score ranges from 0 to 30 points, with a usual cut-off of 24, with lower scores indicating a cognitive decline. As well as MMSE scores, data were gathered on the sociodemographic characteristics of the users and their caregivers, and users’ levels of physical activity during the lockdown.

### 2.4. Structure of the non-pharmacological interventions

Weekly distribution of activities, non-pharmacological interventions (NPI), was established, from Monday to Friday, based on the work plan carried out during all the weeks of the year (Table 1). Although a specific and concrete distribution of the sessions to be carried out was proposed in this protocol, the implementation was flexible and completely adapted to the needs and possibilities of each patient according to the therapist’s judgment. The guidelines of the Department of Health and Social Welfare of the Autonomous Community of Castilla y León, Spain, were followed. Benchmark NPIs were selected that are related to dementia prevention that would have a substantial impact on health care according to WHO guidelines (8, 34). It was recommended to start the day with a cognitive stimulation session that lasted 60 min. We always began by working on orientation to reality, using techniques through which the person becomes aware of his or her situation in time, space, and with respect to his or her person. Subsequently, the session was continued by exercising a different cognitive capacity each day of the week. After the cognitive stimulation, the gerontogymnastics sessions were carried out for 30 min, ending with a series of relaxation exercises Three days a week, after the gerontogymnastics session, the psychomotor skills session was started for approximately 45 min. In the afternoons from Monday to Thursday, two different therapies were worked on, alternating, depending on the day, sessions of activities of daily living (ADLs) (40 min) with sessions of labor therapy (60 min). Two types of ADLs were carried out: (i) Basic activities related to self-care: bathing, dressing, eating, personal hygiene, use of the toilet, toilet training, transfers, transfers, walking and use of stairs; (ii) Instrumental activities tasks aimed at interacting with the environment and generally optional by nature as use of money, medication management, use of public transport, everything related to running the home, shopping. Various types of laborotherapy activities were carried out: (i) clay modeling; (ii) thread branch; (ii) pointillism; (iii) collage with tissue paper; (iv) recoverable with paper. Also, during the rest period on Friday afternoons, they generally used it as free time and recreation without any fixed task.

**Table 1:** Weekly distribution of non-pharmacological interventions (NPI).

Monday	Tuesday	Wednesday	Thursday	Friday
Morning				
Cognitive stimulation (60′)	Cognitive stimulation (60′)	Cognitive stimulation (60′)	Cognitive stimulation (60′)	Cognitive stimulation (60′)
Reality orientation and reminiscences	Reality orientation and attention and praxias	Reality orientation and language, literacy, reading and writing	Reality orientation and memory	Reality orientation and calculus and executive functions
Gerontogymnastic session (30′)	Gerontogymnastic session (30′)	Gerontogymnastic session (30′)	Gerontogymnastic session (30′)	Gerontogymnastic session (30′)
Afternoon				
Activities of Daily Living (40′)	Laborotherapy (60′)	Activities of Daily Living (40′)	Laborotherapy (60′)	Rest

′, minutes.

A team of professionals (Table 2) carried out the NPI in an adult daycare center. All therapies may have the support of nursing assistants/gerocultors.

**Table 2:** Professionals who carry out non-pharmacological interventions (NPI).

Therapy	Therapist
Cognitive stimulation	Psychologist or neuropsychologist
Gerontogymnastic	Physiotherapist
Psychomotricity	Specialist in psychomotricity (psychologist, physiotherapist or occupational therapist)
Laborotherapy	Occupational therapist or animator sociocultural
Activities of daily living	Occupational therapist

### 2.5. Data collection

Two study investigators (D.F.-L. and J.S.-C.) examined electronic medical records and performed specific tests (MMSE) designed for this study. Sex, age, highest educational level (secondary education, higher education, or university), marital status (married or partner) of dementia’s patients and caregivers were included. Sleep quality (poor, fair, good, or excellent) was assessed employing Sleep Quality Scale (SQS), and physical activity during COVID-19 confinement [not physical activity, low level of physical activity (≤3 h), or high level of physical activity (>3 h)] were collected for dementia’s patients. Also, relationship to primary caregiver (spouse or partner, daughter), occupational status (worker) household members (≥3), increase in hours of care, and good health was evaluated by Health-Related Quality of Life Scale of caregivers were registered (Table 3).

**Table 3:** Characteristics of the study’s participants.

Characteristics	Patient full cohort (*n* = 80)	CI (95%)
Gender, n (%)		
Female	57 (71.2)	60.1–80.3
Age (years), mean (SD)	84.8 (6.5)	83.3–86.2
Highest education level, n (%)		
Secondary education	7 (8.9)	4.0–17.3
Higher education or a university	7 (8.9)	4.0–17.3
Marital status, n (%)		
Married/Partner	34 (42.5)	31.7–53.8
Sleep quality^1^, n (%)		
Poor	8 (10.0)	4.5–18.4
Fair	21 (26.2)	17.1–26.7
Good	28 (35.0)	24.7–46.2
Excellent	21 (26.2)	17.1–36.7
Physical activity during COVID-19 confinement, n (%)		
Not physical activity	59 (73.7)	56.0–71.2
Low level of physical activity^2^	21 (26.2)	18.8–34.0
High level of physical activity^3^	0 (0)	0–2.6
Characteristics	Caregiver full cohort (*n* = 80)	CI (95%)
Gender, n (%)		
Female	58 (72.5)	61.7–81.6
Age (years), mean (SD)	58.26 (11.2)	55.75–60.7
Marital status, n (%)		
Married or partnered	45 (60.8)	49.3–71.9
Relationship to primary caregiver, n (%)		
Spouse or partner	12 (15.0)	8.3–24.7
Daughter	63 (78.8)	68.3–86.6
Highest education level, n (%)		
Secondary education	22 (29.3)	19.6–40.6
Higher education or a university	39 (52.0)	40.6–63.5
Occupational status, n (%)		
Worker	58 (80.6)	69.7–88.7
Household members, n (%)		
≥3	29 (36.2)	26.0–47.5
Increase in hours of care^4^, n (%)	66 (83.5)	73.7–90.6
Good health^5^, n (%)	73 (91.2)	82.9 (96.0)

n, participants; values are expressed as mean ± standard deviation for quantitative variables and as frequency (percentage) for categorical variables; SD: standard deviation; CI: confidence interval. ^1^Sleep Quality Scale (SQS), Likert-type scale. ^2^≤ 3 h per week. ^3^> 3 h per week. ^4^Question Self-reported. ^5^Health-Related Quality of Life Scale.

### 2.6. Statistical analysis

To describe the characteristics of the sample means and standard deviations were used for continuous variables and frequencies and percentages for categorical variables, respectively and the 95% confidence intervals were calculated for the corresponding population values. We fitted a mixed model to the change in MMSE score between the periods before and after home confinement. For each individual, an event was defined as having experienced an acceleration in the decrease in MMSE score during the period of home confinement compared to the previous period. The mixed model has been used to adjust for the rate at which each individual worsens. Subsequently, fitted a logistic regression to see which factors appear to be related to more acceleration in the worsening in MMSE of the individuals. We calculated odds ratios (ORs) for this event as a function of the factors analyzed and adjusted the predictive model for this event using logistic regression. All the explanatory variables, related to the patient and his/her caregiver, were candidates to take part in the final logistic regression model. The variables included in this model were selected following the protocol proposed by Hosmer and Lemeshow. Based on the model estimated, we calculated the sensitivity and specificity of the prediction rules. The statistical significance level was set at *p* ≤ 0.05. Data analysis was performed using R statistical package v4.0.2 (35). The statistical significance level was set at *p* ≤ 0.05.

## 3. Results

### 3.1. Sample and characteristics

**Figure 1:**
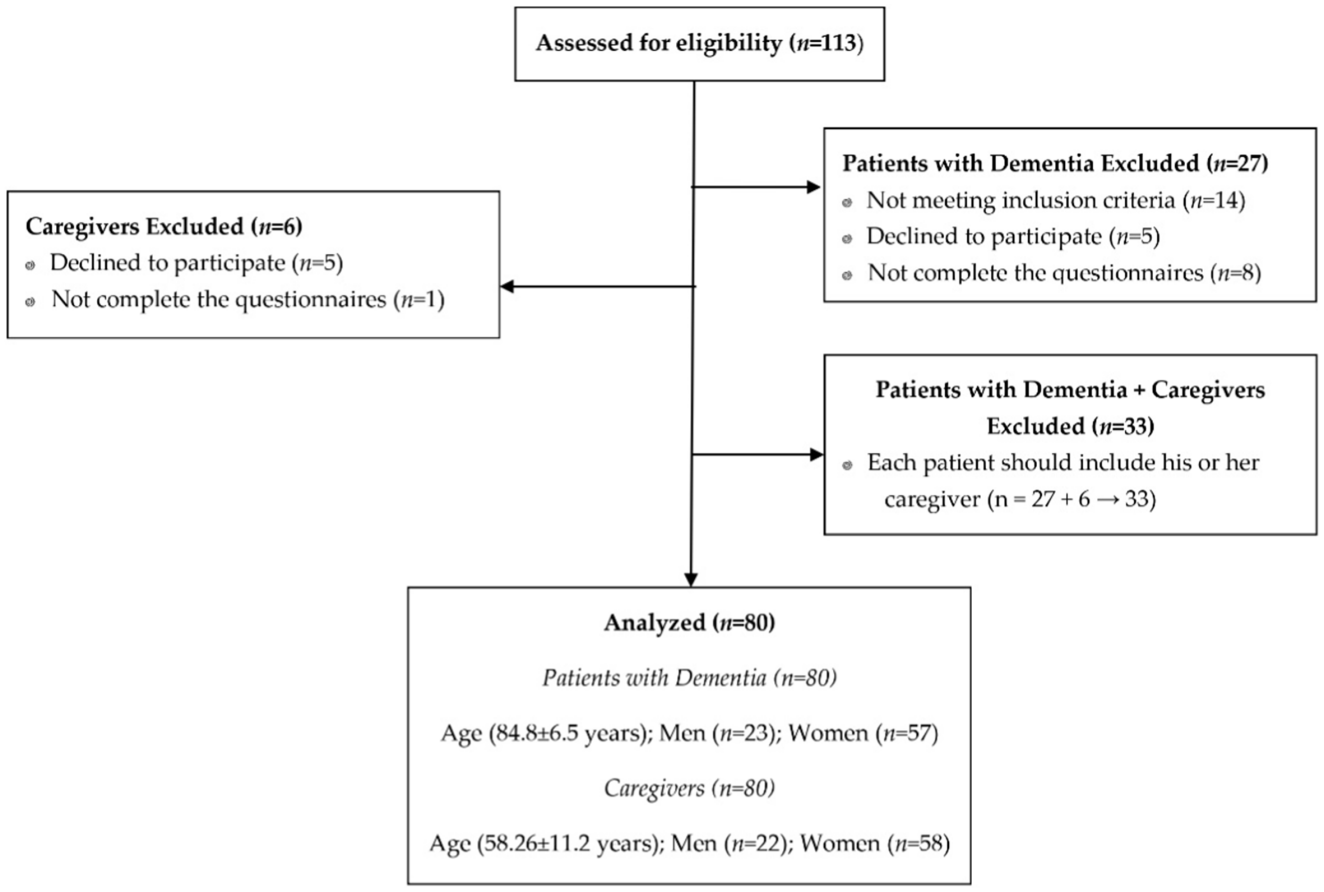
STROBE flowchart for recruitment.

Among the 113 eligible participants invited to participate in the study, six (5.3%) caregivers were excluded (five refused to participate and one did not complete the questionnaires), and 27 (23.9%) dementia patients were excluded (14 not meeting inclusion criteria, five declines to participate and eight did not complete de questionnaire). Therefore, the remaining 80 patients and caregivers fulfilled the inclusion/exclusion criteria and comprised the final study sample (Figure 1).

The patient sample consisted of 57 (71.2%) women, and 58 (72.5%) women were in the caregiver’s group. The mean age of dementia patients was 84.8 ± 6.5 and of caregivers 58.26 ± 11.6. The highest educational level was college or university education of 7 (8.9%) dementia patients and 39 (52.0%) of the caregivers. Thirty-four (42.5%) patients and 45 (60.8%) caregivers were married or had a partner. Regarding sleep quality, 8 (10.0%) patients had poor sleep quality, and 28 (35.0%) patients had good sleep quality. Fifty-nine dementia patients had no physical activity. A total of 63 (78.8%) caregivers were daughters, 58 (80.6%) were externally employed, and 73 (91.2%) caregivers reported good health (Table 3).

### 3.2. Mini-Mental State Examination analysis

Table 4 describes the relationship between accelerated MMSE score loss during home confinement and individual characteristics. A 0.4-point decrease in MMSE score/month (IQR = 1.4) was observed in day center users over the period of home confinement versus a 0.1-point decrease/month before this period (IQR = 0.3) (*p* = 0.038). Notably, this translated to a > 10-point decrease in MMSE score/year in 33.8% of participants during lockdown versus only 5.5% before this period (*p* < 0.001).

**Table 4:** Relationship between accelerated loss in MMSE score during home confinement and individual characteristics.

	Patient full cohort (*n* = 80)				
	OR^1^	*p*-value^1^	OR^2^	CI (95%)^2^	*p*-value^2^
Gender					
Female	1.02	0.975			
Age (for each 10-year increase)	1.11	0.680			
Level of education (without normal/primary/secondary/higher education)	0.63	0.267			
Marital status Married or with a partner	1.26	0.608			
Subject sleep quality^3^ (poor/fair/good/excellent)	1.03	0.892			
Physical activity during COVID-19 confinement Without physical activity	2.12	0.159			
Loss in MMSE score/year Before confinement	0.66	0.001	0.51	0.35–0.75	<0.001
MMSE score Start of confinement (estimated)	1.07	0.040	0.89	0.79–1.00	0.05

	Caregiver full cohort (*n* = 80)				
	OR^1^	*p*-value^1^			
Gender					
Female	0.85	0.753			
Marital status					
Married or with a partner	1.99	0.163			
Age (for each 10-year increase)	1.10	0.690			
Relationship to primary caregiver					
Daughter	0.84	0.757			
Level of education					
(Without normal/primary/secondary/higher education)	0.78	0.421			
Occupational status					
Worker	0.81	0.728			
Household members					
≥3	0.55	0.210			
Increase in hours of care^4^, n (%)	1.33	0.643			
Good health^5^, n (%)	1.04	0.960			

MMSE, Mini-Mental State Examination; OR, odds ratio; MMSE, Mini-Mental State Examination; Multi, multivariate. ^1^Univariate analysis. ^2^Multivariable analysis. ^3^Sleep Quality Scale (SQS), Likert-type scale. ^4^Question Self-reported. ^5^Health-Related Quality of Life Scale. Estimated OR corresponding to explanatory variables with multiple levels are related to the change between consecutive levels. They estimate the risk associated with the posterior level compared to the previous one.

**Figure 2:**
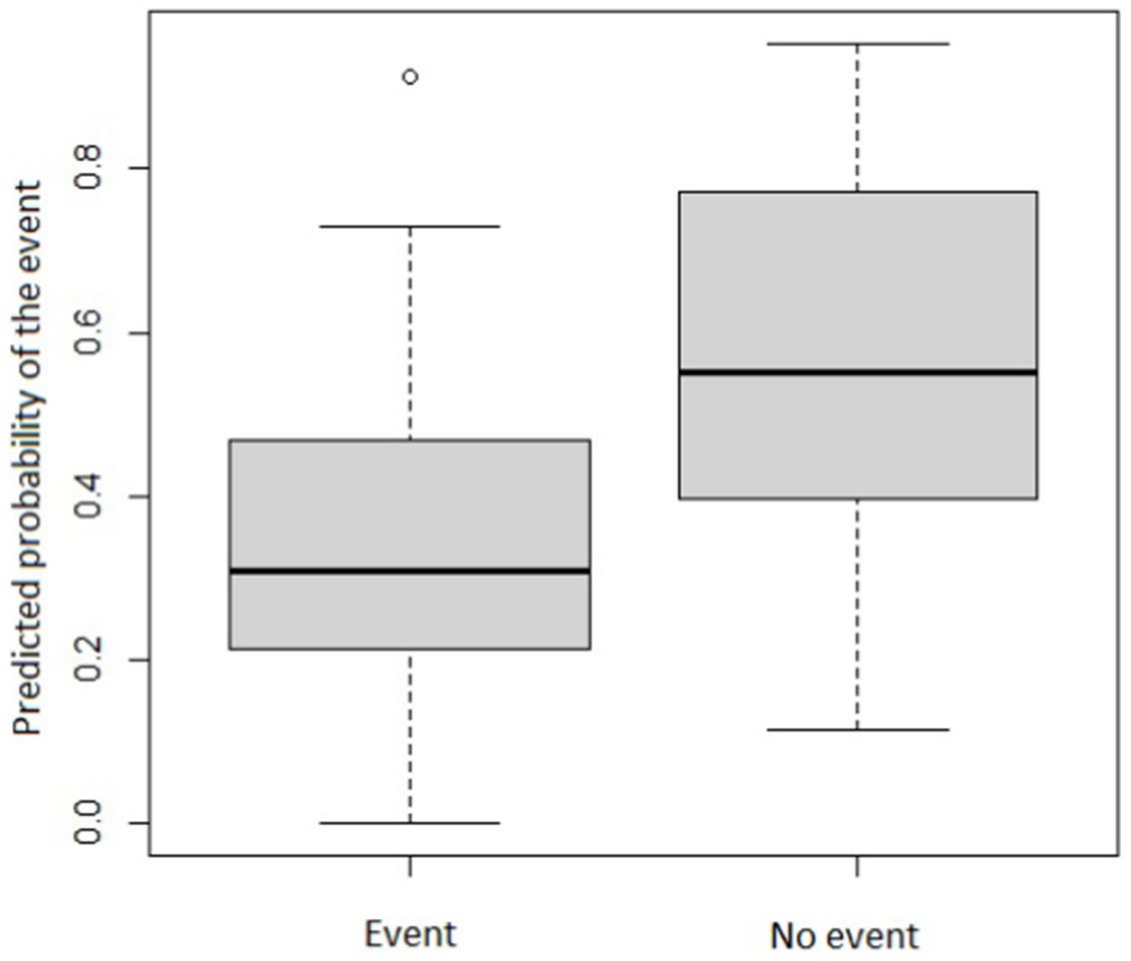
Probability of accelerated cognitive decline in individuals who did and did not experience this event. °*p*-values ≤ 0.05 to be statistically significant.

We defined the event of interest, namely, an acceleration in cognitive decline, understanding this to be indicated by individuals experiencing a decrease in MMSE score over a year (in MMSE/year units) that was >1.5-points larger than the decrease expected under “normal” conditions corresponding to the period before home confinement when they were regularly attending the day center. Table 4 shows the ORs and *p*-values corresponding to the relationship between the onset of the event, which is an acceleration in cognitive decline, and the factors analyzed which were the individual characteristics of users and caregivers. The amount of decline in the previous period as assessed using the MMSE score (loss in MMSE score/year) and the absolute MMSE score just before the start of the home confinement were found to be independent factors for predicting an acceleration in cognitive decline. A prediction rule based on these two characteristics had a sensitivity of 69% and a specificity of 70.6% for predicting the onset of this event. Further, decreases in MMSE scores were larger in patients who had been less active. However, not statistically significant (*p* < 0.05) associations were found between the individual characteristics of caregivers and the onset of the event. Figure 2 shows the distribution of the probabilities of the event predicted by the estimated classification rule in individuals who did and did not show acceleration in cognitive decline.

## 4. Discussion

Using the MMSE questionnaire, our pilot study assessed the impact of discontinuing NPIs on cognitive impairment in patients with dementia during the 3-month COVID-19 lockdown. These dementia patients did not attend the ADC of the “Leonese Association of Dementia Patients” (Leon, Spain) to perform the therapeutic work protocol of NPI, due to restrictions related to the COVID-19 pandemic. Our initial hypothesis was partially fulfilled, with a substantial acceleration of the cognitive decline in patients following a markedly lower MMSE questionnaire score during the confinement period, than in the pre-pandemic COVID-19 period. In our study MMSE scores decreased at a rate of more than 10 points/year in one-third (33.8%) of people during the period of home confinement, in contrast to only 5.5% of those who experienced that rate of decline in the months immediately pre-COVID-19. Furthermore, the prediction rule developed considering independent factors for predicting an acceleration in cognitive decline (loss in MMSE score/year before home confinement and the absolute MMSE score just before the start of this lockdown) had a sensitivity of 69% and a specificity of 70.6%.

Our results could show that NPIs, according to WHO guidelines (8, 34), are a very useful tool for stimulating the abilities of people with dementia, slowing down the development of the disease, and improving the person’s remaining abilities (26, 36, 37). For this reason, the interruptions (COVID-19 quarantine) would affect the performance of the person in their daily life, which will result in a worse state of mind and cognition, a decrease in self-esteem, finally achieving a lower quality of life for the person with dementia (38). The COVID-19 pandemic negatively affected older adults’ engagement in behaviors that promote mental health due to the protective measures implemented and the resulting social isolation (39). This was mirrored by previous outbreaks of SARS and Middle East Respiratory Syndrome (MERS) that demonstrated that quarantine has negative effects on mental health, with an increase in psychiatric symptoms (40). Vernuccio et al. (41) have reported a significant increase in the incidence and severity of psychological disorders in people with dementia due to COVID-19 quarantine. Loneliness, social isolation and loss of routine activities could be an important cause of increased anxiety and depression, which would intensify cognitive impairment during the pandemic, due to patients’ inability to adapt to new living conditions and inability to continue daily activities, such as NPIs (39, 41). ADCs for the elderly are a type of daycare center oriented toward continuity and long-duration care. These ADCs are staffed by a team of highly qualified personnel who attend to the social, and health (comprises mental health) needs of frail elderly people, including patients diagnosed with dementia (42). It is known that the attendance of these patients at ADCs have a dual, physical, and psychological positive effect due to the substantial reduction of problems such as social isolation, depression, and anxiety (43).

In the “Leonese Association of Dementia Patients” ADC, we perform cognitive stimulation therapy (CST) 5 days per week for 1 h daily. The CST would be key to maintaining the cognitive functioning of patients with dementia (44). In this sense, Coen et al. (45) demonstrated that CST substantially improved MMSE scores compared to patients without CST. In addition, patients with early stages of dementia have reported beneficial effects on their mental state and delayed cognitive decline post-CST program (46). CST could enhance the effects of cholinesterase inhibitors, which are prescribed to slow cognitive decline, in reducing the rate of cognitive decline (47) and improving scores on the MMSE. These results suggest that CST is one of the important NPI treatment strategies for patients with dementia.

However, Kallio et al. (48) have reported that CST did not affect global cognition in persons with mild to moderate dementia. Thus, another cause of the decrease in the MMSE score could be the social distancing of confinement by COVID-19. Isolation has been associated with a negative impact on physical and mental health, manifesting this in patients with dementia with higher levels of depression, hopelessness, and cognitive impairment (49). ADCs have “green spaces” in the open air such as gardens or fields, as the ADC of the “Leonese Association of Dementia Patients,” allows social interaction with other users and day center staff, in addition to carrying out programmed NPI activities outdoors. Also, country walks help concentration, as they require greater attention than any other environment (50). As a result, all this could help maintain or improve cognitive abilities.

We observed a relationship, not statistically significant, between having less physical activity and a decrease in the MMSE score. The low level of physical activity, for 3 months of COVID-19 confinement, of the day center patients could lead to reduced cognitive stimulation, provided by exercise, compatible with cognitive impairment (51). The physical activity (gerontogymnastics) program would be able to modulate substrates associated with neuroplasticity (neurotrophic signaling, neurogenesis, inflammation, stress response, and antioxidant defense) in the brain, responsible for cognitive and cerebral improvement (52).

The practice of physical activity in outdoor recreational spaces allows patients to reduce internal tension. This eliminates hypertension, increases the mental well-being of the elderly, and modulates their stress levels (53). The elderly who has seen their stress levels reduced see improvement in pathologies such as depression or conditions such as anxiety, reduced vulnerability and loss of skills, self-esteem, and cognitive impairment (54). Therefore, all this impacts a palpable improvement in the physical condition of older adults, which is always an advantage for their mental health to improve. The reduction of stress and the practice of exercise improve the synthesis and release of dopamine in the brain associated with the mesolimbic pathway, and with the calcium-calmodulin dependent pathway, respectively. This increase, in dopamine, modifies and/or affects brain function, potentially inducing beneficial physiological, behavioral and psychological changes (55, 56).

Family members caring for patients with dementia routinely experience stress, physical impairment, and social isolation (57). Dementia patients are a type of population that was first in and last out of strict and prolonged periods of confinement to prevent SARS-CoV-2 infection. Longer periods of confinement result in more severe neuropsychiatric symptoms that directly affect their caregivers, particularly increased caregiver strain (58). The caregivers, included in our study, showed a substantial increase in their tasks with an optimum state of good health. Adult dementia ADCs provide support and relief to caregivers, showing a positive effect on the relationship between family caregivers and dementia patients (59). NPI-based day interventions give family members a break and have a positive psychological impact, associated with improvements in their social and professional lives (60).

### 4.1. Future scenarios

ADCs have been forced to temporarily close during the COVID-19 pandemic, affecting service delivery. The restrictions that have been implemented in many countries to control the pandemic have also had important neuropsychiatric consequences for patients with dementia (58). For this reason, creative solutions should be proposed to attend to users of ADCs, in future potential home confinements, or for other reasons that prevent the patient with dementia from attending the ADCs. An alternative would be to offer virtual telehealth programs (e.g., support groups, music, and exercise therapy) and “home” visits to support CST efforts and combat loneliness. Through a model based on remote assistance (videoconference and phone calls) for patients, they could maintain the NPIs they had before the pandemic.

Thus, the impact of the COVID-19 pandemic on people with dementia requires (re)thinking to define how we should act, not only in emergency situations, to guarantee a good quality of life for the elderly. Perhaps this implies a radical change in the care paradigm or a more flexible management that knows how to adapt to current health conditions. On the other hand, we must not forget that the support of a health system that accompanies the people, families, and professionals involved is essential. Finally, as a society we should learn from this pandemic to become aware of the impact it has had on the mental health of people who live with a special condition such as dementia.

### 4.2. Limitations and strengths

The authors of this review acknowledge several limitations. Firstly, the small sample size. Secondly, we only assessed cognitive impairment with a single questionnaire, MMSE. Third, a single-center, observational, retrospective, longitudinal epidemiological study was conducted and therefore causality cannot be derived from it because exposure and outcome are evaluated simultaneously. In addition, our study is a single-center study that only included patients with dementia who attended the ADC of the Leonese Association of Dementia Patients (Spain). Therefore, the results we provide, in this pilot study, should be taken cautiously, and given the limitations, we caution against generalizing the results without further investigation. However, we believe that using a single questionnaire allowed us to rapidly reassess the post-COVID cognitive status of patients, to begin implementing our NPIs designed following the WHO guidelines (8, 34), seeking to make them as effective as possible from the outset. In addition, our study was carried out following the STROBE rules (31).

## 5. Conclusion

Our results suggest that, during the 3-month COVID-19 lockdown, dementia patients who typically attend our ADC to receive our NPI program experienced more rapid cognitive decline. The reported declines in MMSE scores reveal a significant acceleration of cognitive decline during the period of inactivity. This could suggest that our NPIs, focused on slowing cognitive decline, are beneficial and, therefore, necessary in patients with dementia.

## Data availability statement

The original contributions presented in the study are included in the article/supplementary material, further inquiries can be directed to the corresponding authors.

## Ethics statement

The studies involving human participants were reviewed and approved by the Ethics Committee of the University of León (Spain) with PI No. 2020–13. The patients/participants provided their written informed consent to participate in this study.

## Author contributions

LS-V, DF-L, and JS-C: conception and design of the study. LS-V and LB-C: data acquisition. AM-I and LS-V: analysis and interpretation of data. DF-L and JS-C: drafting the manuscript and revising it critically for important intellectual content. All authors have approved the version to be published and agreed to be accountable for all aspects of the work in ensuring that questions related to the accuracy and integrity of any part of the work are appropriately investigated and resolved.

## Funding

This work was supported by the Spanish Ministry of Economy and Competitiveness (grant no. MTM2017-86061-C2-1-P) and Department of Education of the Government of Castilla y León and European Regional Development Fund (grant nos. VA005P17 and VA002G18).

## Conflict of interest

The authors declare that the research was conducted in the absence of any commercial or financial relationships that could be construed as a potential conflict of interest.

## Publisher’s note

All claims expressed in this article are solely those of the authors and do not necessarily represent those of their affiliated organizations, or those of the publisher, the editors and the reviewers. Any product that may be evaluated in this article, or claim that may be made by its manufacturer, is not guaranteed or endorsed by the publisher.
